# Charting the molecular landscape of the cell

**DOI:** 10.1016/j.str.2023.08.015

**Published:** 2023-09-11

**Authors:** Hannah Ochner, Tanmay A.M. Bharat

**Affiliations:** 1 Structural Studies Division, MRC Laboratory of Molecular Biology, CB2 0QH Cambridge, UK

## Abstract

Biological function of macromolecules is closely tied to their cellular location, as well as to interactions with other molecules within the native environment of the cell. Therefore, to obtain detailed mechanistic insights into macromolecular functionality, one of the outstanding targets for structural biology is to produce an atomic-level understanding of the cell. One structural biology technique that has already been used to directly derive atomic models of macromolecules from cells, without any additional external information, is electron cryotomography (cryoET). In this perspective article, we discuss possible routes to chart the molecular landscape of the cell by advancing cryoET imaging as well as by embedding cryoET into correlative imaging workflows.

## Introduction

Biological macromolecules exist in crowded environments inside cells, tissues, and organisms. In many cases, biological function is not only associated with the structure of individual molecules, but emerges as a result of the interactions between different molecules.^1,2^ An in-depth understanding of this molecular sociology not only requires high-resolution structural information about individual molecules, but also of their localization and interactions in complex cellular settings.^1,3^ This, in turn, necessitates the accurate mapping of macromolecules and complexes in their functional context, i.e., within the intact cellular environment.^1,4^

A technique ideally suited for the direct visualization of molecules in their biological environment, supporting *in situ* imaging within the cellular context, is electron cryotomography (cryoET).^1,4–8^ By combining information from two-dimensional (2D) projections of the same sample imaged at different tilt angles with respect to the electron beam, a three-dimensional (3D) volume of the sample, termed tomogram, can be computationally reconstructed. Tomograms are 3D snapshots of the specimen, typically at high nanometre resolution, allowing the spatial localization of target macromolecules within the sample. Higher-resolution structural information can be resolved via the averaging of different copies of macromolecules in the tomogram in a process called subtomogram averaging. Furthermore, cryoET supports direct correlation of the tomographic data with information obtained from a variety of associated techniques such as fluorescence microscopy and mass spectrometry (MS).

This combination of cryoET with associated techniques provides a unique opportunity to fulfill the long-standing goal to accurately and comprehensively map the macromolecular landscape of a cell. Ideally, all molecules within the cell could thus be localized and chemically identified in their native environment.

In this perspective article, we review recent developments that have brought the field closer toward this aim and discuss possible directions to be explored in the future. We identify two complementary paths that could contribute to this goal: the direct improvement of tomographic imaging and reconstruction, as well as the use of orthogonal correlative imaging techniques which allow the chemical identification of molecules within the cellular context.

## Direct identification of molecules in tomograms

A tomogram is a reconstruction of a 3D cellular volume and thus, in principle, includes all the relevant spatial information about the target macromolecules. Many macromolecules, however, do not have distinct shapes or appearances that make them stand out at the typical tomographic resolution, making it difficult to distinguish these target macromolecules from the tens of thousands of other macromolecules present in the cell. Macromolecular localization in tomograms is hence directly linked to tomogram quality and resolution, as better tomograms allow distinguishing between more molecular species. Therefore, increasing tomogram quality and resolution is an important future area of development for the field.

There are several intricately interrelated factors that limit tomogram resolution. The most important and fundamental limitation is radiation sensitivity of biological specimens.^9^ Radiation sensitivity leads to restrictions in the usable dose and thus in achievable signal-to-noise ratio, which reduces contrast and complicates the alignment of the tilt series images. An accurate alignment is, however, essential for high-resolution tomogram reconstruction. This limitation is less severe in the imaging of inorganic specimens, where significantly higher doses, and thus resolutions, are possible.^10^ Other resolution-limiting factors are the increased sample thickness of specimens prepared to retain a biologically intact sample state, which correlates with an increasing number of multiply scattered electrons and thus further affects the signal-to-noise ratio, and unavoidable reconstruction artifacts due to missing tilt angles, i.e., the “missing wedge”.

Given that the 3D information contained in a tomogram is retrieved from the information stored in each of the images acquired at different tilt angles, an artefact-free, high-resolution tomographic reconstruction in principle requires imaging at the full angular range, sampled as finely as possible to completely map out the corresponding 3D Fourier space.^11^ As, in practice, both the number of obtainable tilt series images as well as the accessible angular range are limited by radiation damage effects and sample geometry,^1,2^ these factors, especially dose, number of projections, and sample thickness, have to be carefully balanced to obtain high-resolution tomograms.

Given the diversity of interconnected factors currently limiting tomogram quality and resolution, there are several levels on which technical advances could lead to significantly improved tomograms, ranging from improvements in sample preparation and hardware to innovations in data processing and optimisations in the data acquisition process.

## Improvements in sample preparation and microscope hardware

The interaction of the electron beam with the sample can lead to significant beam-induced motion of the target molecules, which is often associated with radiation-related distortions of the ice layer that is not perfectly planar.^12,13^ Specimen movement leads to blurring and inaccurate tilt series alignment and can even induce changes in the specimen during tilt series acquisition, resulting in an irrecoverable loss of resolution.^14^ Thus, tomogram quality and resolution could be significantly improved by the reduction of specimen movement during data collection. On the specimen preparation side, the use of ultrastable gold supports that limit radiation-induced specimen movement^13,15,16^ is a proven method to improve tomogram quality.^14^ Further development in specimen supports is likely to improve tomographic reconstruction and is the subject of research in several laboratories.

In addition, sample thickness is a major limiting factor in cryoET, because increased sample thickness leads to an increase in inelastic scattering events during imaging, resulting in lower signal-to-noise ratios in the collected tilt series images. Preparation of thinned specimens, especially when working with cellular samples, alleviates this problem. Thin samples can be obtained using a variety of techniques that range from targeting thin edges of cells (such as filopodia), to the use of genetic mutants producing smaller cells (sometimes called mini-cells), and to the sectioning of frozen specimens using a diamond knife followed by subsequent imaging (CEMOVIS).^17–21^ In recent years, the adaptation of focused ion beam (FIB)-milling for biological specimens^22,23^ has substantially advanced cryoET imaging. FIB-milling employs an ion beam to ablate material from the specimen above and below the target area, resulting in thin lamellae with a thickness of 100–250 nm containing the sample volume of interest. While FIB-milling can lead to structural damage to the molecules near the surface of the lamellae and thereby might restrict the achievable resolution, in many cases, sample thickness is the more dominant factor causing deterioration of signal-to-noise ratio.^24^ FIB-milling is thus a controlled technique for producing thin samples suitable for cryoET with negligible damage to the interior of the specimen, which is applicable to a wide range of cellular systems.^22–34^ Recent advances in automation of lamellae milling^23^ have dramatically increased the throughput of this promising method. Further automation of this important technique will result in higher quality cellular tomograms for macromolecular mapping.

Complementing the developments in sample preparation, additional advances on the microscope hardware side that have the potential of improving tomogram data include imaging at helium temperature, which has been shown to reduce radiation damage to the specimen,^35^ and chromatic aberration correction^36,37^ which can enhance the imaging of thicker samples in which inelastic scattering processes are significant. The chromatic aberration of the objective lens results in the inability to focus electrons of different energies (i.e., different wavelengths) onto the same focal plane. Thus, inelastically scattered electrons, which lose energy during the interaction with the specimen, are not correctly focused. These electrons either increase noise in the micrographs or are discarded by the use of an energy filter, not providing any useful information about the specimen. Chromatic aberration (C_c_) correction would correct for this and hence allow inelastically scattered electrons to contribute to the image and thereby improve the signal-to-noise ratio of tomograms, which are inherently restricted by the dose limitation characteristic of biological specimens.^36,37^

Developments in detector technology and sensitivity can further improve the quality of tomographic imaging. Direct electron detectors allow for faster data acquisition by acquiring rapid image sequences instead of single images, thereby reducing specimen movement within tilt images, and supply images with substantial information transfer even at the sampling limit, i.e., the Nyquist frequency.^2,38,39^ In addition, an increased imaging speed boosts throughput, especially when combined with faster tilt schemes. Some examples of such tilt schemes include continuous tilting of the goniometer combined with a rapid acquisition of tilt frames,^40^ fast-incremental tilt schemes^41^ or parallelized acquisition of multiple areas of the specimen using beam image shifts.^42^ As the use of direct electron detectors has been a major factor in enabling electron cryomicroscopy-based structural biology up to atomic resolution, further improvements of detector performance, for example by an increased detective quantum efficiency and a higher accuracy in the subpixel localization in electron counting, could be an important contribution to increasing tomogram quality and resolution.^43,44^

## Advances in data acquisition and tilt series data processing

Another route toward tomogram enhancement is the optimization of data acquisition and data processing schemes. Data collection and processing are tightly connected, and therefore must be considered together for any tomographic experiment. Improvements in these can boost resolution and reduce artifacts induced by physical limitations of the imaging process discussed previously.

The tilt scheme used for acquiring the tomographic data has a significant effect on the quality of the tomogram, because it determines in which angular region the highest quality data can be obtained, as the number of obtainable projections is strictly dose-limited. Indeed, only few tilt images contain high-resolution information required for high-resolution structural biology, since this information quickly gets lost due to radiation damage. Thus, the implementation of a dose-symmetric tilt scheme,^45^ which begins acquisition on the untilted—and hence thinnest—sample, and gradually employs higher and higher tilts while alternating between positive and negative tilt angles, has been shown to maximize the obtainable resolution, as estimated by subtomogram averaging.^46^ Such a scheme is now standardly used in many cryoET applications. Advances in achievable resolution offered by the dose-symmetric tilt scheme could be further improved by combining it with other modified tilt schemes which have shown the potential for an overall improvement of tomographic reconstruction, such as equally sloped tomography (EST).^11^ EST is a tilt scheme that aims at the optimization of the mapping between the set of tomographic projections, which are described in polar coordinates, and the reconstructed object described in Cartesian coordinates, in order to reduce artifacts and improve the quality of the reconstruction. This is realized by using pseudopolar fast Fourier transforms, which provide an exact mapping from a Cartesian grid to a pseudopolar grid featuring equally sloped lines rather than the equally angled lines of a polar grid. These mathematical considerations can be implemented into the tomographic imaging process by combining pseudopolar Fourier transforms with a tilt scheme that yields equally sloped tomographic projections in pseudopolar coordinates. Hence, instead of acquiring projections at equal increments of the tilt angle (*θ*), EST uses a set of tilt angles determined by equal steps of the slope of the tilt angle (tan *θ*). This scheme, combined with an iterative oversampling algorithm, has been shown to improve tomogram resolution as well as dose-efficiency in several applications, achieving better than 4 Å-resolution for electron tomography of inorganic specimens.^10,47–51^ Advances on the data acquisition level can thus substantially improve tomographic imaging, especially if they have the potential for increasing dose efficiency. Another potential method to increase tomogram quality is the use of dual-axis tomography, which collects tilt series in two orthogonal tilt axes. Dual-axis tomography has been shown to reduce wedge-related artifacts and thus increase the quality of tomographic re-constructions.^52–54^ For radiation-sensitive samples, however, employing a dual-axis scheme has to be carefully balanced with dose considerations, and applying this method is cumbersome, involving grid rotations in the microscope, leading to limited reported applications in the last few years.

Tomogram quality can additionally be improved by using computational solutions that correct for remaining artifacts. Alignment accuracy could for example be increased by integrating multi-particle refinement schemes in subtomogram averaging,^55^ which improve tilt series alignment by correcting for beam-induced motion. Other studies have reported improvements by employing center-of-mass-based alignment schemes.^10^ In addition, mathematical concepts such as compressed sensing,^56,57^ which exploits the sparsity of a signal to reduce the number of necessary measurements to recover it, may be employed to reduce artifacts in cryoET reconstructions,^58–63^ and can allow improvements in signal-to-noise ratio and resolution of the resulting tomograms without the need for additional data collection.

## Improvements in template matching techniques and tomogram processing

In order to draw conclusions about a molecule of interest, it must first be localized within the densely packed cellular environment observed in tomograms. A technique that has been successfully employed to aid with particle identification within tomograms is template matching.^64,65^ Template matching computationally identifies target molecules within a tomogram by comparing voxels within the tomographic volume with a template. The choice of template depends on the *a priori* knowledge available about the target molecule. A typical example would be lowpass-filtered features of a known atomic structure of the molecule of interest.^64,65^ Additional steps, such as segmentation or denoising, can further improve structure identification and reduce the number of false positives detected by the template matching process.^6,66,67^ This automated way of localizing targets within a tomogram provides a visualization of the spatial distribution of the macromolecules of interest, hence subsequently applying the method with a range of templates could delineate the 3D arrangement of the different cellular components. Template matching is thus a valuable asset in assembling a complete map of the cellular landscape.

**Figure 1:**
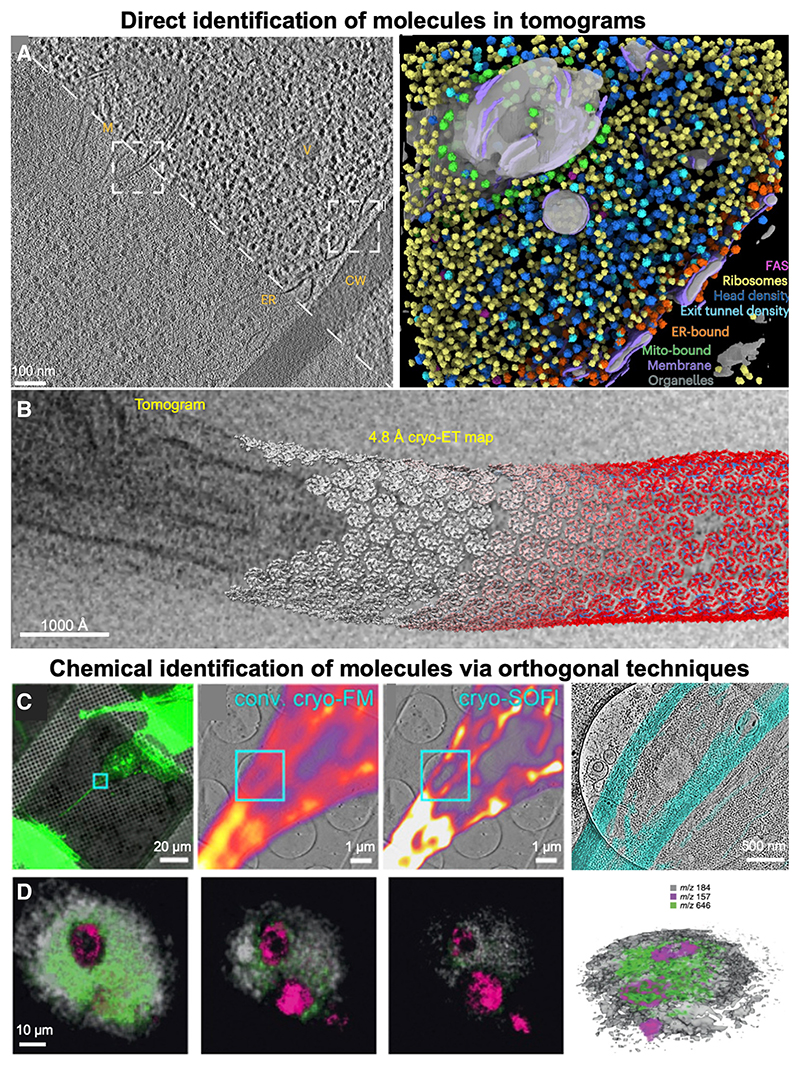
Techniques for localizing macromolecules within the cellular environment (A) Mining molecular patterns in tomograms of cellular volumes using neural network-based picking. Left: 2D slice of a tomogram of *Schizosaccharomyces pombe*, raw data (below dashed line) and contrast-enhanced by an amplitude equalization filter (above dashed line). Discernible organelles are labeled: mitochondrion (M), vesicle (V), endoplasmic reticulum (ER) and the cell wall (CW). Right: Macromolecular localization prediction for the cellular volume by the convolutional neural network-based software DeePiCt. Figure adapted from ref.^72^ (B) Employing high-resolution maps obtained by subtomogram averaging for localizing macromolecular structures in cellular tomograms. Slice through the tomogram of a *Caulobacter crescentus* cell overlaid with copies of the 4.8 Å-resolution structure of the S-layer, composed of repeating copies of the RsaA protein, from subtomogram averaging in the refined locations. Figure adapted from ref.^83^ (C) Super-resolution cryoCLEM of mammalian cells. Left to right: Overlay of conventional cryo fluorescence microscopy (cryo-FM, green) and reflected light (gray) overview images of mammalian cells with Dendra2-Lifeact-labeled actin; overlays of tomogram slices of the area indicated in the left image with conventional cryo-FM (center) and with cryogenic super-resolution optical fluctuation imaging (cryo-SOFI); overlay of corresponding tomogram sections with actin-containing volumes (cyan). Figure adapted from ref.^93^ (D) 3DFIB-SIMS imaging of a rat alveolar macrophage cell incubated with the drug amiodarone. Left to right: Sequence of time-of-flight SIMS images of the sample as the cell is milled by a Bi^3+^ ion beam showing the distribution of phosphocholine marker (gray), nuclear marker (magenta), and amiodarone (green) in different layers of the specimen, and 3D rendering of the corresponding volume. Figure adapted with permission from ref.^114^

Recent developments in template matching include *de novo* template generation and template-free pattern detection, i.e., the localization of target molecules from the tomographic data alone, without any external references or templates.^6,68,69^ Other developments are the inclusion of deep learning- and neural network-based approaches to pattern mining and template generation, which have significantly advanced the accuracy and the speed with which patterns can be detected in tomograms,^68–73^ especially when combined with the computational prediction of potential protein-protein interactions.^74–76^ For example, macromolecular location prediction within a tomogram using the convolutional neural network-based software DeePiCt led to the identification of macromolecular complexes of varying shapes and sizes (Figure 1A).^72^

The ability to locate individual macromolecules opens up another important possibility of tomogram resolution improvement via subtomogram averaging structure determination, which we discuss briefly for completeness. In recent years, the resolution of macromolecular structures obtained from tomographic data has been pushed beyond previous limits by employing subtomogram averaging.^14,77–83^ Subtomogram averaging requires the extraction of subvolumes containing copies of the target molecule from a tomogram and their subsequent 3D alignment and averaging analogously to single-particle analysis for cryoEM.^1,79^ The averaging process enhances contrast, and as the orientations of the individual molecular copies differ with respect to the missing wedge, anisotropy in the reconstruction is reduced. Subtomogram averaging can thus significantly improve the resolution of the resulting molecular structure determined from target molecules in the cellular environment and also reveal the orientation and interactions of the molecules, which are important for building the molecular picture of the cell, including macromolecules in the cytoplasm or molecules forming ordered superstructures in cells (Figure 1B). Subtomogram averaging has been successfully applied to a range of macromolecules including ribosomes,^84^ virus particles,^85,86^ bacterial or archaeal surface layers,^87,88^ proteasome complexes^6^ and membrane proteins.^89^ As subtomogram averaging relies on target identification for subvolume extraction, subtomogram averaging and template matching are highly synergistic methods that can be combined to yield efficient structure determination workflows for macromolecules in the cellular context.

In summary, cellular tomographic data not only contains structural information about classes of molecules, it also contains information regarding the localization of said molecules, as well as their orientation relative to one another. As the cellular environment is densely packed with a range of different molecules, identifying molecules of interest is crucial both for subtomogram averaging and for studying interactions of molecules within their native environment.

## Chemical identification of molecules in cells using orthogonal techniques

While the direct improvement of tomogram quality and resolution will be crucial for the creation of a full molecular atlas of the cell, correlation of cryoET data with molecular localization data from different imaging approaches, especially methods exhibiting chemical sensitivity, has the potential of significantly contributing to this goal.

A correlative imaging technique that has been successfully applied to a range of cellular processes is cryo-correlative light and electron microscopy (cryoCLEM).^21,90^ CryoCLEM combines analysis by cryoET with a fluorescent light microscopy imaging step performed at cryogenic temperatures. Cryo-light microscopy provides a map of all fluorescently tagged molecules, which can then directly be correlated with cryoET data^91^ (Figure 1C). This process of correlation is often aided by the use of fiducial markers visible in both light and electron microscopy.^91^ As cryoET itself is not a chemically sensitive technique, prior identification of the target molecules via fluorescent tagging permits molecular localization in tomograms.^21^ Molecular identification and localization in cryoCLEM can further be improved by utilizing advances in light microscopy resolution such as super-resolution techniques^92,93^ (Figure 1C) or single-molecule localization microscopy.^94–96^ Given that not all molecules of interest can be tagged fluorescently, alternative tagging strategies could be used to facilitate molecular localization within the cell. As metals appear at higher contrast in electron micrographs, metal-containing molecules such as gold-antibody conjugates,^97^ metallothionein,^98^ and ferritin^99^ could be used as tags in electron microscopy imaging. Within the framework of electron microscopy, electron energy loss spectroscopy (EELS) and cryo-scanning transmission electron microscopy (cryo-STEM) can also provide insights into the chemical identity of the imaged objects.^100–103^ In the case of EELS, the unique energy loss spectrum of each element is exploited for chemical identification, while in cryo-STEM, the scattering angle of the incident electrons is a function of the atomic number of the chemical element, which can be utilized for identification.

To gain enhanced chemical sensitivity without the need for tagging, cryoET can be combined with MS-based techniques. When studying macromolecules and their interactions in the cellular context, one option is the correlation of high-resolution spatial data obtained from cryoET with cross-linking MS.^104,105^ As cross-linking MS provides information about expected interactions via the formation of additional bonds between residues in close proximity, this data can help with the interpretation of unexplained densities or provide guidance when searching for the relevant macromolecules and their interaction partners in the tomogram volume.^105^ The prediction of protein-protein interactions can also significantly be aided by artificial intelligence-based methods,^74–76^ which can be integrated into correlative workflows including template matching and cross-linking MS. Specific interactions, especially those involving small molecules or ligands, can also be explored using native MS,^106–108^ which can further guide interpretation of tomograms. In addition, native MS-based techniques could be integrated into the sample preparation process for electron microscopy imaging of purified biological samples,^109,110^ pointing toward possible fruitful future developments in combined MS and electron microscopy workflows.

Another MS-based technique with potential for correlative imaging with electron microscopy is focused ion beam secondary ion mass spectrometry (FIB-SIMS). In FIB-SIMS, the secondary ions created by focused ion beam milling of the sample are analyzed using a mass spectrometer,^111–113^ thus the technique directly combines spatial information gathered by FIB/SEM with chemical information from MS. This approach hence has the advantage of allowing nearly simultaneous spatial and chemical imaging. As material is continuously ablated during the FIB-SIMS process, the method also has access to the 3D sample volume. 3D imaging by FIB-SIMS has been successfully applied to identify small molecules in tissue specimens (Figure 1D). While FIB-SIMS is an established technique for elemental analysis in materials science,^111,113^ the FIB-SIMS analysis of biological samples is more complex, as larger molecules need to be identified via their fragmentation pattern. However, developments in recent years such as the use of gas cluster ion beam sources and advanced mass spectrometers have facilitated an increase in the obtainable spatial and mass resolution as well as the identification of larger molecules in biological samples.^112,114–116^ Combined with lamella preparation and cryoET, FIB-SIMS is a promising technique, which could provide information about both the chemical identity and the spatial localization of molecules within their native cellular environment.

## Conclusion

The feasibility of structural biology *in situ* has been demonstrated by multiple developments in cryoET discussed in this article, culminating in the high-resolution structure determination of a growing number of macromolecules directly from the cellular environment. While some parts of the cell, such as the nucleus, as well as molecular complexes with low abundance, will be very challenging to map, the continuous improvement of the methods employed for this purpose could, in the future, contribute to the generation of a full atlas of the cellular landscape. The two approaches outlined in this article, namely the overall improvement of tomogram resolution and the chemical identification and localization of molecules in the cell using correlative techniques, are both promising strategies for mapping the molecular landscape of the cell. The two approaches are harmonious and the best results will likely be obtained if they are employed in parallel. In summary, the future of structural biology is bright; as techniques for whole cell investigations mature, the possibility of charting the molecular landscape of the cell will soon become a reality.
